# GNE myopathy with premature ovarian failure: Case report and review of the literature

**DOI:** 10.1016/j.ymgmr.2025.101240

**Published:** 2025-07-01

**Authors:** Shangyi Yang, Jine Yang

**Affiliations:** Department of Neurology, Hebei Cangzhou Hospital of Integrated Traditional Chinese Medicine and Western Medicine, Cangzhou 061000, China

**Keywords:** Nonaka myopathy, GNE myopathy, *GNE* gene, Premature ovarian failure, Extra-muscular manifestations

## Abstract

GNE myopathy (GNE-M) is an ultra-rare disease characterized by muscle weakness in the extremities. The main etiology is that a pathogenic variation in the *GNE* gene leads to a reduction in sialic acid synthesis. However, whether it is associated with premature ovarian failure (POF) isunknown. Here we report a case of GNE-M with premature ovarian failure (GNE-M-POF). The clinical data of a case of GNE-M-POF in our hospital were collected. The historical trajectory, molecular features, diagnosis and treatment were analyzed retrospectively, and the literature was reviewed. Over the course of 12 years, the patient presents with a slow and progressively worsening clinical manifestation. She was amenorrheic at the age of 35 and never conceived. Her calf muscles, and some of her thigh muscles were severely atrophied, leaving her unable to walk on her own. POF may be an extra-muscular manifestation of GNE-M, and further research is needed to verify the association.

## Highlights

• GNE myopathy(GNE-M) is an ultra-rare disease, which begins with muscle weakness.
• Some extra-muscular manifestations associated with GNE-M have been reported.
• There are few descriptions in the literature of GNE-M involving reproduction, such as premature ovarian failure (POF), and whether it is associated with POF is unknown.
• Herein, we report a case of GNE-M with POF.

## 1 Introduction

GNE myopathy (GNE-M), which was formerly known as Nonaka myopathy (NM), is an ultra-rare autosomal recessive distal myopathy caused by a biallelic variant in UDP-*N*-acetylglucosamine 2-epomerase/*N*-acetylmannosamine kinase (GNE) [1]. The estimated worldwide prevalence of GNE myopathy is 1 to 9 per million [1].In the early stage of the disease, patients mainly present with weakness in the distal extremity, and rimmed vacuoles can be seen in muscle biopsy, which has been referred to as distal myopathy with rimmed vacuoles and hereditary inclusion-body myopathy type 2 (IBM2) [2]. Study has found that NM and IBM2 are both caused by variants in the *GNE* gene, which was defined as GNE-M in 2014 [3]. The classical clinical manifestation of the disease begins with muscle weakness at the distal extremity, which has been confirmed by a number of studies [4,5]. Recently, as atypical clinical manifestations, some extra-muscular manifestations associated with GNE-M have been reported, such as idiopathic thrombocytopenia, cardiac involvement, respiratory involvement, psychological disorders, and sleep apnea syndrome [6,7]. However, there are few descriptions in the literature of GNE-M involving reproduction, such as premature ovarian failure (POF). Herein, we report a case of GNE-M with premature ovarian failure (GNE-M-POF) and investigate GNE-M-POF by reviewing its historical trajectory, molecular features, diagnosis and treatment.

## 2 Case description

A 43-year-old female patient was admitted to Hebei Cangzhou Hosipital of Integrated Traditional Chinese and Western Medicine in 2024 due to menstrual disorder for 12 years and weakness of limbs for more than 10 years. She had her initial menstrual disorder in 2012 at the age of 31 and amenorrhea at the age of 35. Two years later, she was found to have an abnormal walking posture and gradually developed weakness in her left lower limb and fingers in both hands. The concentration of creatine kinase (CK) was 307 U/L. Magnetic resonance imaging (MRI) of the thighs showed selective atrophy of the muscles, especially the posterior muscle groups and the adductor muscle groups. Electromyography revealed myopathic changes. In 2019, she was diagnosed with GNE-M combined with POF. After that, the patient felt that the strength of the muscles in the limbs gradually decreased, especially in the lower limbs.

## 3 Diagnostic assessment

Neurological examination revealed that the patient's bilateral thenar muscles and the left calf muscles were obviously atrophied and her muscle strength of the extremities was abnormal. The strength of her deltoid and biceps brachii muscles was grade 4 and the strength of her forearm and hand muscles was grade 3 according to the Medical Research Council (MRC) criteria. In her lower extremities bilaterally, the proximal muscles were categorized as grade 3 and the distal muscles were categorized as grade 2 according to the MRC criteria. She could turn her head because of MRC grade 3 strength of the sternocleidomastoid muscles; however, neck flexion was unavailable at a supine position. She walked with a long stride. In the early stage of the disease, she manifested as a steppage gait with hip circumduction in her left leg. As the disease progresses, she presented with a waddling gait. She could not walk in a straight line, nor could she walk on tiptoes and heels.

**Fig. 1:**
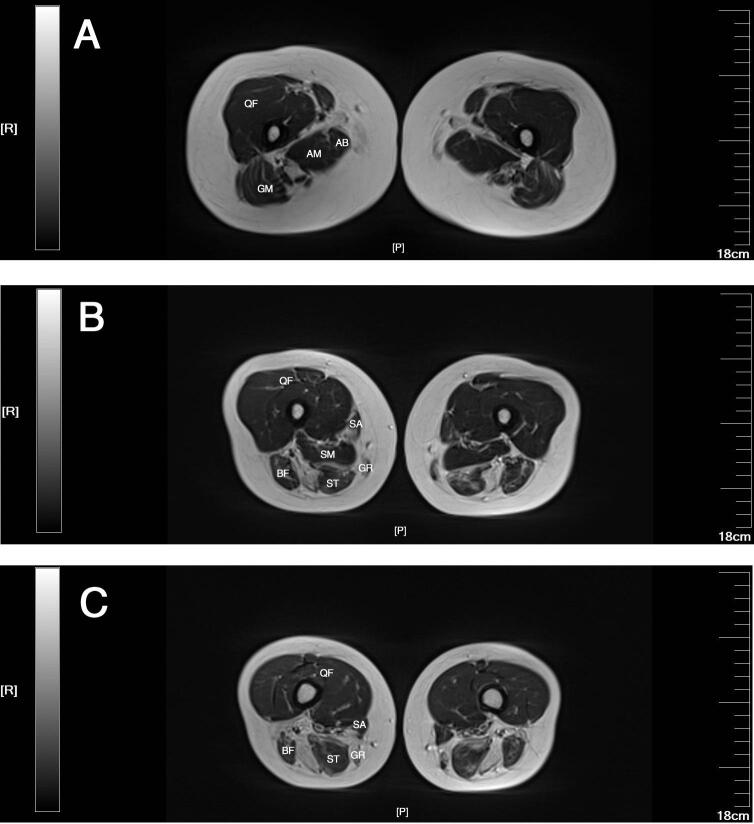
Axial T2-weighted magnetic resonance imaging (MRI) of the thighs at different planes, respectively the proximal (A), middle (B) and distal (C) planes. MRI of the adductor magnus (AM), adductor brevis (AB), gluteus maximus (GM), biceps femoris (BF), semitendinosus (ST), gracilis (GR), semimembranosus (SM) and sartorius (SA) showed different degrees of atrophy and fatty replacement. The quadriceps femoris (QF) were relatively preserved.

MRI of the thighs showed selective atrophy of the muscles, especially the posterior muscle groups and the adductor muscle groups. MRI of the adductor magnus, adductor brevis, gluteus maximus, biceps femoris, semitendinosus, gracilis, semimembranosus and sartorius muscles showed different degrees of atrophy and fatty replacement. The quadriceps femoris muscles were relatively preserved (Fig. 1). Unfortunately, MRI of the lower leg muscles was not performed.

Needle electromyography of bilateral tibialis anterior, medial gastrocnemius and semitendinosus muscles, as well as left interosseus I and left flexor carpi radialis muscles showed denervation potentials, narrow and small motor unit potentials (MUPs), and early recruitment., with sparing of the vastus medialis and biceps brachii muscles. Nerve conduction studies were normal. It revealed myopathic changes.

**Fig. 2:**
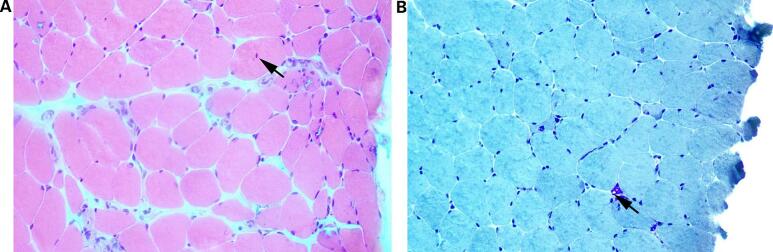
Muscle pathology findings of the patient in optical microscopy. (A) HE staining ×400: the muscle fibers varied in size, a few muscle fibers were mild to moderate atrophy and there were centranucleated muscle fibers (arrow); (B) MGT staining ×400: the muscle fibers varied in size and rimmed vacuoles (arrow) scattered in a few muscle fibers.

The pathological findings of the right quadriceps biopsy revealed myogenic changes, suggesting marginal vacuolar myopathy (Fig. 2). Two heterozygous variants of the *GNE* gene NM_001128227.2: c.620 A > T (p.Asp207Val) and NM_001128227.2: c.1009C > T (p.Arg337*) were detected by a whole-exome sequencing of genomic DNA extracted from the peripheral blood of the patient (Table 1). The reproductive examination revealed abnormal levels of reproductive hormones (Table 2). Gynecologic ultrasound showed normal uterus, small bilateral ovaries with diameters of 2.01 cm and 1.92 cm, respectively, and no sinus follicles. Pituitary MRI reported no abnormalities.

**Table 1:** Whole exon gene detection of the patient and her family member.

Gene	Zone	Reference sequence	Location	cDNA level	Protein level	Status	Variation classification
GNE^a^	9p13	NM_001128227.2	Exon3	c.620 A > T	p.(Asp207Val)	Heterozygosis	Pathogenic variation
GNE^a^	9p13	NM_001128227.2	Exon5	c.1009C > T	p.(Arg337*)	Heterozygosis	Pathogenic variation
GNE^b^	9p13	NM_001128227.2	Exon3	c.620 A > T	p.(Asp207Val)	Heterozygosis	Pathogenic variation
GNE^b^	9p13	NM_001128227.2	Exon5	c.1009C > T	p.(Arg337*)	Heterozygosis	Pathogenic variation
GNE^c^	9p13	NM_001128227.2	Exon5	c.1009C > T	p.(Arg337*)	Heterozygosis	Pathogenic variation

GNE, glucosamine (UDP-*N*-acetyl)-2-epimerase/*N*-acetylmannosamine kinase; “a” represents the patient; “b” represents her sister; “c” represents her mother; Asp, Aspartic Acid; Val, Valine Acid; Arg, Arginase Acid; “*” represents termination codon.

**Table 2:** The reproductive hormone level of the patient.

Detection time	FSH (mIU/mL)	E2(pg/mL)	PRL(ng/mL)	AMH(ng /mL)
August 2019	58.25	10.31	6.47	<0.01
October 2019	62.08	8.97	5.86	<0.01

FSH, follicle-stimulating hormone; E2, estradiol; PRL, prolactin; AMH, anti-Mullerian hormone.

**Fig. 3:**
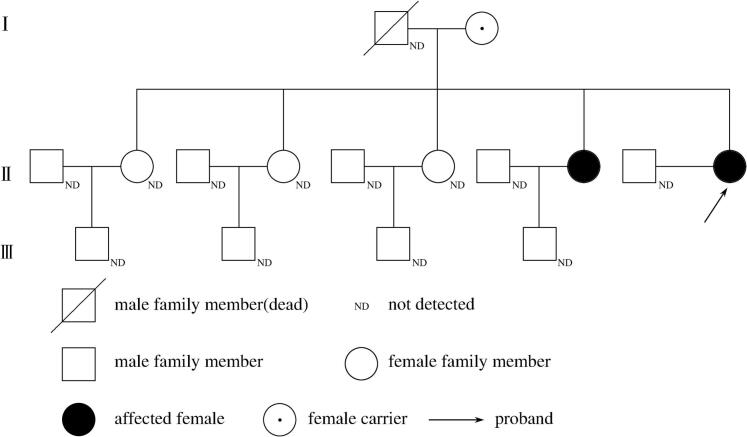
The pedigree of three generations of the patient's family.

The patient has five older sisters. Of her five older sisters, only her fourth sister has left lower limb weakness and carries the same genetic variants of the *GNE* gene as the patient. Since her fourth sister had regular periods and gave birth to a child, she was reluctant to undergo the examinations related to ovarian function, such as hormone levels, imaging tests, etc. Therefore, we cannot accurately tell if her ovarian function is normal. The patient's three other sisters did not demonstrate similar clinical manifestations, had regular periods and gave birth to children. The patient's parents also did not demonstrate similar clinical manifestations. Her father died of lung cancer and her mother is very well though she carries a heterozygous pathogenic variant NM_001128227.2:c.1009C > T (p.Arg337*) of the *GNE* gene (Table 1).There is a pedigree of three generations of the patient's family (Fig. 3).

She was given medication, including neurotrophic drugs, endocrine modulating drugs such as vitamin B1 tablets, mecobalamine tablets, and symptomatic management. She was not receiving targeted sialic acid therapy. She was advised to maintain a healthy lifestyle, such as moderately increasing food intake high in protein, sialic acid and moderate exercise to avoid fatigue. The patient's condition is that her hands can hold objects, but she cannot take care of himself. Her calf muscles and some of her lower leg muscles are severely atrophied, leaving her unable to walk on her own. She was never conceived without using contraception.

## 4 Discussion

GNE-M is a distal limb myopathy caused by *GNE* gene variants with autosomal recessive inheritance [1]. *GNE* gene variants leads to the inability of some important glycoproteins or glycolipids to sialate, which is the main pathogenic mechanism of this disease [1]. GNE-M was formerly known as NM which was first described independently by Nonaka in 1981 as distal myopathy with rimmed vacuoles [8] and by Argov and Yarom in 1984 as rimmed vacuole myopathy sparing the quadriceps [9]. At present, the disease has been uniformly defined as GNE-M [3].

GNE-M usually occurs in young people, mostly between 20 and 40 years old, with an average of 28.9 years old [1]. The course of the disease progresses relatively slowly and the average time from onset to the need for a wheelchair being 11.9 years [10].The typical clinical symptoms of this disease are slowly worsening muscle weakness and muscle atrophy at the distal extremity [4]. As the disease progresses, it gradually involves proximal lower extremities, girdle muscles, axial muscles and upper limb muscles [11]. Most patients do not have lesions in the quadriceps muscle, but a few patients have different degrees of quadriceps weakness in the early stage and even involve myocardium and respiratory muscles [6]. Furthermore, some patients also present with some atypical symptoms, such as a patient with chronic axial low back pain and right buttock pain [12], a patient with a complaint of left foot drop [13], patients with sleep apnea syndrome and patients with thrombocytopenia [6,7]. However, there are no relevant reports in cases involving reproductive system and presenting as POF.

Muscle biopsy is the most important way to diagnose the disease [1]. Its pathological findings under microscopy are marginal vacuoles in the muscle fibers, basophilic particles at the edge of the vacuoles, and no monocyte infiltration [11]. Under electron microscopy, it is found that these rimmed vacuoles are actually fine filamentous inclusions in the nucleus of muscle fibers with high acid phosphatase activity and positive lysosomal marker proteins, suggesting that they are related to autophagy [14]. It should be noted, however, that marginal vacuoles are also seen in diseases other than GNE myopathy and should be distinguished [11].

GNE-M is caused by a biallelic mutation in *GNE* gene and the main etiology is that a pathogenic variant in the *GNE* gene leads to a reduction in sialic acid synthesis [15]. Sialic acid is widely distributed in the body, usually located at the end of the sugar chain of glycoproteins and glycolipids, and regulates some important biological processes [16]. It can affect the synthesis of sialic acid and cause hypo-sialylation of key proteins, damage of cytoskeletal network, apoptosis of myotome tissues and cells, thus leading to a series of clinical and pathological changes [17]. Studies have shown that when a gene is mutated, it does not affect the expression of the gene, but rather the activity of the enzyme the gene encodes [18]. In vitro studies further confirmed that different types of *GNE* gene variants lead to different changes in enzyme activity [19,20]. Therefore, it can be inferred that the reason why different gene variation types cause different clinical phenotypes may be due to the different enzyme activities, which provides clues for further exploring the relationship between genotype and phenotype [21]. Moreover, when the *GNE* gene is mutated, it may affect enzymes that regulate the functional activity of other cells in addition to sialic acid [22]. On the other hand, its variation types and hotspots vary by human race and geographic location. Met743Thr is the most common variation site in the Middle East, Asp207Val, Cys44Ser and Val603Leu are common in Japan, while Asp207Val is predominant in China [1].

The treatment of the disease includes rehabilitation training, dietary modification, and alternative therapies aimed at maintaining muscle function as much as possible and delaying disease progression [11]. There is still no approved drug or standard treatment for the disease [11]. However, with the development of molecular biology, especially the *GNE* gene, the efficacy of gene therapy can be expected [23].

POF refers to amenorrhea in women before the age of 40 years, detected gonadotropin levels greater than 40 mIU/mL, decreased estrogen levels, and accompanied by symptoms of varying degrees of low estrogen levels [24]. The causes of POF include genetic, immune, infection, environmental and medical factors, etc., but most of them are primary and are associated with gene function loss, variation or polygenic heterozygous variation [25,26]. Genetic variation is an important factor contributing to POF [27]. This patient has no risk factors such as immunity, infection, drug induction, etc., which may be caused by genetic factors. According to her genetic testing report, we found two mutation sites in her *GNE* gene on chromosome 9p13: c.620 A > T (p.Asp207Val) and c.1009C > T (p.Arg337*). Unfortunately, we could not find any studies on their association with POF. A study from China found that 27 % of female patients with GNE myopathy experienced rapid deterioration during pregnancy or after delivery [28]. Another study from Japan showed a higher frequency of threatened abortion in women with GNE myopathy during pregnancy [29].

POF is highly heterogeneous genetically, and it has been confirmed that many genes are involved in the pathogenesis of POF, mainly including X chromosomal and autosomal abnormalities and gene defects. Among which, the abnormality of Fragile X mental retardation 1 gene (FMR1) [30] and the variants of galactosemia-related genes are the common genetic variants of POF [31]. Variants in genes associated with galactosemia include various types. Type 1 galactosemia is due to variants of the galactose-1-phosphate uridylyltransferase (GALT) gene mapped on chromosome 9p13 and is the most frequent galactosemia form [32]. The first enzyme of the galactose metabolic pathway is galactose mutarotase (GALM) an aldose epimerase that catalyzes the reversible interconversion between β- and α-D-galactose. GALT is involved in the subsequent metabolic step [33]. GALT converts Gal-1-P into glucose-1-phosphate (Glc-1-P) with the formation of UDP-galactose (UDP-Gal) from UDP-glucose (UDP-Glc) by a “ping-pong” mechanism. UDP-Glc is essential for the enzymatic activity of GALT [34]. In the presence of GALT deficiency, accumulated galactose-1-phosphate can also activate the pyrophosphorylase pathway (in particular UDP-glucose pyrophosphorylase) resulting in the UDP-Glc and uridine diphosphate (UDP)-hexose deficiency. The activation of the above-mentioned alternative pathways contributes to increased oxidative stress and endoplasmic reticulum stress, impaired glycosylation, and altered signaling pathways [35].

Patients with GNE-M inherited in an autosomal recessive manner carry variants in the *GNE* gene which affect the sialic acid synthesis pathway. The *GNE* gene is UDP-*N*-acetylglucosamine 2-epomerase/*N*-acetylmannosamine kinase [15,36] which has the dual function of epitopase and kinase and is independent of each other. It can catalyze UDP-*N*-acetylglucosamine (UDP-GIcNAc) to form the heterotropic isomer *N*-acetylmannosamine (ManNAc) and phosphorylate it to ManNAC-6-P. Abnormal encoding of this enzyme can cause GNE myopathy.

Additionally, we have not found relavernt studies that the GNE expressed directly in ovarian tissue, but a study indicated that GNE is comparatively high expressed in the trophoblast lineage. Since trophoblast cells are closely related to the processes of embryo implantation and pregnancy, and the ovary plays a crucial role in the reproductive process, from this perspective, it can be speculated that there may be some indirect connection between the *GNE* gene and ovarian function [37]. Some studies pointed out that changes in sialylation modification are related to the formation of the tumor microenvironment of ovarian cancer and abnormal sialylation is involved in the metastatic process of ovarian cancer, which in turn affects ovarian function [38,39]. Some researches showed that there is a certain functional connection between GNE and galactose-related enzymes in *Aeromonas hydrophila*, though we have not found relevant researchs that galactosemia-related genes have functional interactions with the patient's GNE mutations [40,41].

Therefore, we speculated that GNE-M may have been involved in the reproductive system, and POF in this patient may be related to the abnormal activity of the enzyme regulating ovarian function caused by different loci and type variants of *GNE* gene. It is necessary to further study its pathogenic mechanism.

## 5 Limitations

The study reports a case of a patient with GNE-M and POF, and we suggest that POF may be an extra-muscular manifestation of GNE-M. However, there are some limitations in our study. One limitation is that it only proposes a hypothesis based on a single case and needs to future verify the causal relationship between GNE variants and POF through multi-center cohorts or gene editing models. Moreover, the patient's fourth sister carries the same GNE mutation and has lower limb weakness, but she was reluctant to undergo the examinations related to ovarian function, such as hormone levels, imaging tests, etc. Therefore, we cannot accurately tell if she has premature ovarian insufficiency. Additionally, of note, the number of trinucleotide repeats in the FMR1 gene was not analyzed and the patient was reluctant to undergo relevant examinations, we thus cannot rule out Fragile X-associated primary ovarian insufficiency.

## 6 Conclusion

GNE-M-POF may be a rare disorder in which POF may act as an extra-muscular manifestation of GNE-M. Its pathogenesis needs further study.

## Publisher's note

All claims expressed in this article are solely those of the authors and do not necessarily represent those of their affiliated organizations, or those of the publisher, the editors and the reviewers. Any product that may be evaluated in this article, or claim that may be made by its manufacturer, is not guaranteed or endorsed by the publisher.

## Patient consent for publication

Written consent for publication has been obtained from the patient. All identifying information has been removed or anonymized to ensure confidentiality.

## CRediT authorship contribution statement

**Shangyi Yang:** Supervision, Resources, Investigation, Formal analysis, Data curation, Conceptualization. **Jine Yang:** Writing – review & editing, Supervision, Investigation, Data curation.

## Ethics statement

Written informed consent was obtained from the individual(s) for the publication of any potentially identifiable images or data included in this article.

## Funding

No funding was received.

## Declaration of competing interest

The authors declare that they have no known competing financial interests or personal relationships that could have appeared to influence the work reported in this paper.

## Data Availability

The original contributions presented in the study are included in the article/Supplementary material, further inquiries can be directed to the corresponding author.
